# Thoracoabdominal aneurysm repair using a four-branched thoracoabdominal graft: a case series

**DOI:** 10.4076/1757-1626-2-7144

**Published:** 2009-07-17

**Authors:** John Kokotsakis, George Lazopoulos, Hutan Ashrafian, Panagiotis Misthos, Thanos Athanasiou, Achilleas Lioulias

**Affiliations:** 1 Second Cardiac Surgical Department, Evangelismos General HospitalIpsilantou Street 45-47, Athens, 106767Greece; 2 Department of Biosurgery and Surgical Technology, Imperial College London, Imperial College Healthcare NHS Trust at St Mary’s HospitalLondonUK; 3 Thoracic Surgery Department, Sismanoglio General Hospital1 Sismanogliou Street, 15126 Marousi, AthensGreece

## Abstract

Revascularization of the visceral arteries during thoracoabdominal aneurysm repair is usually performed sequentially by an anastomosis between a prosthetic graft and an aortic patch. There are immediate operative risks such as bleeding and distortion. In the longer term, aneurysm, pseudo-aneurysm and rupture may occur. These require reoperation and are associated with significant morbidity and mortality.

We present our experience with Crawford IV thoracoabdominal aneurysm repair in four patients, using a prefabricated four-branched graft (Coselli graft). At two years there were no deaths, no complications and no vessel abnormalities on computed tomography. We recommend its use as the graft of choice in young patients with an aortic tissue disorder requiring total resection of the aortic wall at the level of the visceral vessels.

## Introduction

**Figure 1.**
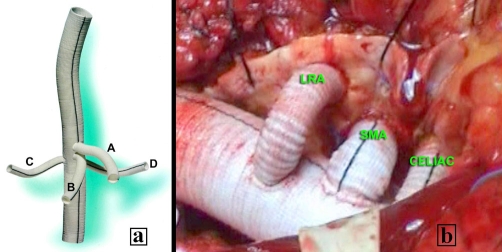
**(a)** Four-branched Coselli graft. **(b)** Intraoperative picture of Crawford Type IV Thoracoabdominal aneurysm repair with the Coselli graft (LRA=left renal artery, SMA=superior mesenteric artery, CELIAC=celiac artery).

The standard surgical treatment for patients with both dissecting and non-dissecting thoracoabdominal aortic aneurysms is an in-situ reconstruction using a tubular Dacron graft. Visceral arteries are attached by a side-to-side anastomosis between the graft and an aortic patch that contains all branches except for the left renal artery, which is usually reimplanted separately [1-4]. Although this approach can reduce operative time, it is not optimal for young patients with connective tissue disease, or those whose residual aortic segment is extensive (due to the distance between vessels), as the residual aortic segment is more prone to dilatation, aneurysm formation and potential rupture during the follow-up period. [5,6]. To address these concerns, a specifically designed multi-branched thoracoabdominal graft has been introduced (Gelweave® Coselli Thoracoabdominal Graft, Vascuteck-Terumo, Renfrewshire Scotland, UK, Figure 1a), originally stemming from a multi-branched transverse aortic arch substitution, [7] with appropriately sized and positioned branches for the celiac, superior mesenteric and both renal arteries.

We present our experience with the Coselli graft in four patients (characteristics in Table 1), emphasizing the ease of its use, indications, technical considerations and graft performance over a 2-year follow-up period.

**Table 1.** Pre-operative characteristics of the four patients

Patient	Age (years) Sex/Ethnicity	Aortic Pathology	Diameter	Operation	Associated risk factors
1	69 female/Caucasian	Chronic dissection	6.2 cm	Elective	HTN, COPD, Bradyarrhythmia
2	68 male/Caucasian	Degenerative	8.2 cm	Elective	HTN, COPD, Right renal artery stenosis, smoker
3	58 male/Caucasian	Degenerative	11 cm	Urgent (acute pain impending rupture)	Dilated Cardiomyopathy, EF: 25%, Renal Dysfunction (Creatinine: 2.5 mg/dl), Smoker
4	72 male/Caucasian	Degenerative	8 cm	Urgent (acute pain impending rupture)	HTN, CAD (CABG × 4), EF: 45%, Smoker

## Case presentation

Access to the thoracoabdominal aorta was provided by thoraco-phrenico-laparotomy and subsequent retroperitoneal exposure. Cardiopulmonary Bypass (CPB) was used in two patients (3 and 4), for after load reduction of the left ventricle due to their compromised cardiac function, while the other two patients (1 and 2) were managed without circulatory support (clamp and sew technique).

The lower descending thoracic aorta was clamped and transected at the level of the diaphragm. The aneurysm was opened longitudinally; back bleeding from the distal aorta was controlled with a balloon catheter. Renal preservation was achieved by direct infusion of 4°C lactated Ringer’s solution with 25 gm/l of mannitol and 1 gm/l methylprednisolone into the renal artery ostia. Low dose dopamine and furosemide were routinely used for renal protection. Cold blood was infused in celiac and superior mesenteric artery for visceral protection in the two CPB supported patients. After proximal anastomosis was performed, side branches of the graft were attached to the celiac, superior mesenteric, right and left renal artery ostia (Figure 1b). To minimize ischemic time of the abdominal organs, blood flow was restored after completion of each side branch anastomosis. The graft was finally anastomosed on the aortic bifurcation. Cross-clamp time ranged from 58 to 75 min.

We consider as important the following technical points to facilitate its use:

1. Perform the proximal anastomosis first and use an occlusion balloon catheter for the distal aorta to have a bloodless operative field.
2. Use the black line of the graft for orientation and keep the length of the branches as short as possible.
3. The use of the smallest applicable size of the Coselli graft results in an easier proximal and distal anastomosis.
4. Anastomose the celiac axis and the superior mesenteric first and then the renal vessels.

**Figure 2.**
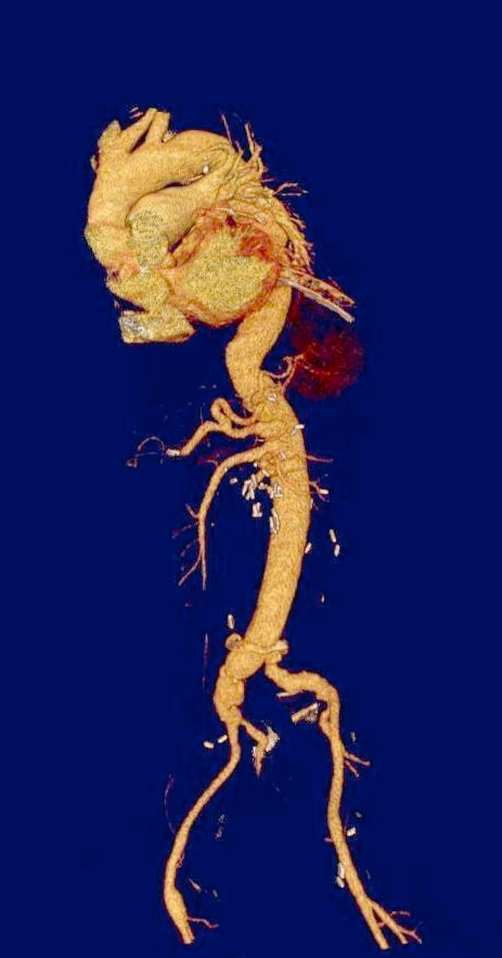
Postoperative Computed Tomographic Angiogram following Crawford Type IV Thoracoabdominal aneurysm repair using the Coselli graft.

There were no in-hospital deaths. Patient 1 required prolonged ventilatory support (4 days) and permanent pacemaker implantation. Transfusion requirements were less than 4 units of packed red blood cells and 4 units of fresh frozen plasma for all patients. Patients 2 and 3 had moderate elevation of creatinine, but none of them required hemodialysis. All abdominal organs showed normal function. Computed tomographic angiography before discharge revealed normal contrast filling of the four side branches without any torsion or compression. At two-year follow-up there was no evidence of aneurysm or pseudoaneurysm formation at the level of visceral vessel anastomoses (Figure 2).

## Discussion

Using the traditional Crawford technique for thoracoabdominal aneurysm repair, the reported incidence of patch aneurysm formation can be as high as 7.5% and can be higher (17%) in Marfan patients [2,3,5,8]. Reoperation is technically demanding due to adhesions in approaching the patch. This carries a significant morbidity and mortality as a result of significant blood loss and renal failure, and is associated with a significant risk of death.

In view of it prefabricated four-branched design, the Coselli graft limits visceral artery patch aneurysm formation and has the added advantage of flexibility, allowing it to adapt to different anatomical needs (distance between visceral vessels). The surgeon decides on the sequential order of anastomosis, allowing for perfusion of each of the major visceral organ, which is not available with the traditional technique. Furthermore, the Coselli graft may save follow-up costs by decreasing complications related to the residual aortic patch thereby reducing the number of readmission and reinterventions.

The disadvantages identified, firstly include the extra time required to complete the four individual anastomoses compared to a single patch anastomosis. Secondly, the orientation and spacing of the branches are fixed and thus may not suit the particular need of each patient, therefore some extra care is required to apply appropriate graft orientation and tailor the appropriate length of the four branches.

In conclusion, we feel that the separated graft technique is a valuable alternative to the classic patch reimplantation technique, as it permits sequential artery reattachment and earlier restoration of blood flow. Furthermore, it eliminates pseudoaneurysm formation and permits the creation of tension free anastomoses obviating early bleeding. We therefore recommend its use as the graft of choice in young patients with an aortic tissue disorder requiring total resection of the aortic wall at the level of the visceral vessels.

## Abbreviations

CAD: coronary artery disease
CABG: coronary artery bypass grafting
COPD: chronic obstructive pulmonary disease
CPB: cardiopulmonary bypass
EF: ejection fraction
HTN: hypertension
